# Extract of *Polygonum cuspidatum* Attenuates Diabetic Retinopathy by Inhibiting the High-Mobility Group Box-1 (HMGB1) Signaling Pathway in Streptozotocin-Induced Diabetic Rats

**DOI:** 10.3390/nu8030140

**Published:** 2016-03-03

**Authors:** Eunjin Sohn, Junghyun Kim, Chan-Sik Kim, Yun Mi Lee, Jin Sook Kim

**Affiliations:** Korean Medicine Convergence Research Division, Korea Institute of Oriental Medicine, Daejeon 34054, Korea; ssen4022@kiom.re.kr (E.S.); dvmhyun@kiom.re.kr (J.K.); chskim@kiom.re.kr (C.-S.K.); candykong@kiom.re.kr (Y.M.L.)

**Keywords:** diabetic retinopathy, high-mobility group box-1, receptor for advanced glycation end products, nuclear factor-kappa B

## Abstract

High-mobility group box-1 (HMGB1) is a well-known pro-inflammatory cytokine. We aimed to investigate the effect of the ethanol extract of the root of *P. cuspidatum* (PCE) on retinal inflammation in diabetic retinopathy. PCE (100 or 350 mg/kg/day) was administered to diabetic rats for 16 weeks, and hyperglycemia and body weight loss developed in the diabetic rats. The retinal expression levels of HMGB1 and receptor for advanced glycation end products (RAGE) and the activity of nuclear factor-kappa B (NF-κB) in the retina were examined. Additionally, a chromatin immunoprecipitation assay was performed to analyze the binding of NF-κB binding to the RAGE promoter in the diabetic retinas. The levels of HMGB1 and RAGE expression, NF-κB activity, and NF-κB binding to the RAGE promoter were increased in the diabetic retinas. However, treatment with PCE ameliorated the increases in HMGB1 and RAGE expression, and NF-κB activity in the retina. In addition, in diabetic rats, retinal vascular permeability and the loosening of the tight junctions were inhibited by PCE. These findings suggest that PCE has a preventative effect against diabetes-induced vascular permeability by inhibiting HMGB1-RAGE-NF-κB activation in diabetic retinas. The oral administration of PCE may significantly help to suppress the development of diabetic retinopathy in patients with diabetes.

## 1. Introduction

Diabetic retinopathy (DR) causes adult vision loss and blindness and is among the principal microvascular complications in diabetic patients. Strong evidence suggests that continuous low-grade inflammation is primarily involved in the pathogenesis of diabetic retinopathy [1]. High-mobility group box-1 (HMGB1) is involved in the pathogenesis of diabetic microvascular complications, plays an important role in the inflammatory response, and its pro-angiogenic processes are closely associated with diabetic retinopathy [2].

HMGB1 is an architectural chromatin-binding nuclear protein that has been implicated as a mediator of both non-infectious and infectious inflammatory diseases [3]. Necrotic cell death can result in the passive leakage of HMGB1 from cells when the protein is no longer bound to DNA, which induces an inflammatory response and promotes tissue repair and angiogenesis [4]. Extracellular HMGB1 functions as a pro-inflammatory cytokine that interacts via receptor for advanced glycation end products (RAGE), and the downstream signaling pathways of RAGE play key roles in the cellular response to diabetic retinopathy [5]. HMGB1 also activates the transcription factor nuclear factor kappa B (NF-κB) to generate an inflammatory response that disturbs the retinal vascular barrier, the blood retinal barrier (BRB) [6]. Thus, the amplification of inflammation and angiogenesis can be mediated by the increased secretion of HMGB1 and the increased expression of its associated receptors. Under diabetic conditions, HMGB1 is known to act as a pro-inflammatory cytokine that is involved in many diabetic complications such as retinopathy and nephropathy. El-Asar *et al.* demonstrated that HMGB1 levels are increased in the retina of diabetic mice [7], and our previous study suggested that hyperglycemia-induced HMGB1 release induces renal injury in diabetic rats [8].

*Polygonum cuspidatum* (*P. cuspidatum*) is the dried root of *P. cuspidatum* Sieb. et Zucc. (Polygonaceae). *P. cuspidatum* is called “Hojang-geun” in Korea, “Huzhang” in China, and “Itadori-kon” in Japan, and it has long been widely used as a folk medicine in Korea, China, and Japan. In Korea, this plant has been used to treat allergenic inflammation [9,10] and is commonly added to daily foodstuffs with a slightly sour taste in China and Japan [10,11]. In traditional Chinese medicine, *P. cuspidatum* has been used as a medicine for several conditions, including diabetes and its complications and inflammation, and as an antiviral agent [12]. In particular, this plant has been used in pre-clinical and clinical practice as an effective agent that promotes anti-inflammation and lipid regulation in various diseases in China and Japan [10,13]. Recent studies have shown that *P. cuspidatum* has an anti-inflammatory effect in rheumatoid arthritis and hepatitis models by inhibiting C-Reactive Protein (CRP) and inflammation-related liver injuries [11,14]. Although *P. cuspidatum* has been used as a medicine to treat diabetes, no clear underlying mechanism has been proposed for this activity. Recently, there have been reports that *P. cuspidatum* exerts a preventative effect against diabetic nephropathy [15]; however, the effect of *P. cuspidatum* on diabetic retinopathy has not been explored. Therefore, the purpose of this study was to investigate the mechanism of its protective effect on diabetic retinopathy in STZ-induced diabetic rats.

## 2. Materials and Methods

### 2.1. Ethics Statement

All experiments were conducted in accordance with the National Institutes of Health (NIH) Guide for the Care and Use of Laboratory Animals and the ARVO Statement for the Use of Animals in Ophthalmic and Vision Research. In addition, the experimental protocol was approved by the Institutional Animal Care and Use Committee (approval number: HH109101). Euthanasia was performed by deeply anesthetizing the rats with intraperitoneal injections (pentobarbital sodium 60 mg/kg) followed by perfusion with saline prior to tissue collection. All efforts were made to minimize animal suffering.

### 2.2. Plant Material and Preparation of PCE

The root of *Polygonum cuspidatum* (*Polygonum cuspidatum* Sieb. et Zucc.) was commercially obtained from Jung-dong, a herbal drug market in Daejeon, Korea, in November 2008. The obtained plant was identified by Prof. Ju Han Kim in the Department of Life Sciences, Gachon University. After drying the root of *P. cuspidatum* (6.8 kg) at room temperature (RT), the plant was ground and then extracted with 100% ethanol (3 × 36 L) at RT for three days. The extracts of the root of *P. cuspidatum* were concentrated *in vacuo* at 40 °C to generate lyophilized PCE (580 g). A voucher specimen was deposited in a herbarium for future reference (specimen number: POCU1e).

### 2.3. HPLC Analysis of PCE

HPLC-grade acetonitrile and water were obtained from Fisher Chemicals (Fair Lawn, NJ, USA) and J.T. Baker (Phillipsburg, NJ, USA), respectively. Analytical-grade acetic acid was obtained from Wako (Tokyo, Japan). HPLC analysis was performed using an Agilent 1200 HPLC instrument (Agilent Technologies, Santa Clara, CA, USA) equipped with a binary pump, vacuum degasser, autosampler, column compartment, and diode array detector (DAD). The column used was a Spherex 5 C18 (5.0 µm, 4.6 × 250 mm, Phenomenex, Torrance, CA, USA). The mobile phase was a mixture of solvent A (acetonitrile) and solvent B (water with 0.1% acetic acid). A linear gradient elution was performed from 97% to 70% of solvent B in 40 min, from 70% to 30% of solvent B in 20 min, and from 30% to 0% of solvent B in 5 min, followed by washing and reconditioning the column. The column temperature was maintained at 30 °C. Analysis was performed at a flow rate of 1.0 mL/min and monitored at 290 nm. The injection volume of the sample was 10 µL.

### 2.4. Induction of Diabetic Animals and Experimental Design

Seven-week-old male Sprague-Dawley rats were injected with streptozotocin (STZ, 60 mg/kg, i.p., Sigma-Aldrich, St. Louis, MO, USA). One week after STZ injection, a blood sample was obtained from the tail vein. The control rats received vehicle (0.01 M citrate buffer, pH 4.5), and the diabetic rats (blood glucose level >250 mg/dL) were divided into four groups (*n* = 8 per group). PCE was dissolved in 0.5% w/v carboxyl methylcellulose (CMC) solution and used as the vehicle. Two groups of STZ-induced diabetic rats received a daily gastric gavage of PCE (100 or 350 mg/kg), and the other two groups received the same amount of vehicle via gavage for 16 weeks. In traditional Korean practice, the usual dosage of the plant is approximately 20 g of the raw plant for a 60 kg human body and is administered once per day. PCE dosages were given orally in the laboratory animal experiments to study its medicinal effects. The powder dosage was calculated based on the human equivalent dosage of the raw plant. After 16 weeks of PCE treatment, the non-fasting or fasting blood glucose levels were measured using an automated analyzer (Hitachi Technology, Tokyo, Japan), and body weight was monitored daily.

### 2.5. Measurement of Retinal Vascular Permeability

After anesthesia was induced using zolazepam (Zoletil, Virbac, Carros, France), 100 mg/kg of fluorescein isothiocyanate-bovine serum albumin (FITC-BSA, Sigma, MO, USA) in sterile PBS was injected into the left ventricle of the experimental rats. The tracer solution was allowed to circulate for 10 min, and then one eye was enucleated. The retinas were dissected, flat-mounted onto a glass slide, and viewed using fluorescence microscopy (BX51, Olympus, Tokyo, Japan).

### 2.6. Immunohistochemical and Immunofluorescence Staining

Immunohistochemistry was performed as previously described [4]. Retinal tissues were fixed in 10% formaldehyde and embedded in paraffin, and 4 μm thick sections were prepared. The primary antibodies used were mouse anti-albumin (Sigma, St. Louis, MO, USA), mouse anti-HMGB1 (Abcam, Cambridge, MA, USA) and rabbit anti-RAGE (Sigma, St. Louis, MO, USA). After an incubation in CAS blocking solution (Zymed Laboratories, San Francisco, CA, USA) for 30 min, the sections were incubated in primary antibodies against HMGB1 and RAGE overnight at 4 °C and then washed three times with PBS. To detect HMGB1 and RAGE in the retinal sections, the slides were prepared using an Envision kit (DAKO, Carpinteria, CA, USA), and immunoreactivity was visualized using a 3,3′-diaminobenzidine tetrahydrochloride peroxidase substrate kit and red-alkaline phosphatase kit (AEC) (DAKO, CA, USA). For immunohistochemistry, nuclear staining was performed by incubating the slides in hematoxylin solution. Immunofluorescence staining was performed on the trypsin-digested retinal vessels. The eyes were enucleated from the rats, and the retinas were isolated. After fixation in 10% formalin for two days, the retinas were incubated with trypsin (3% in sodium phosphate buffer containing 0.1 M sodium fluoride to inhibit DNAse activity) for 60 min. The vessel structure was isolated from the retinal cells by gently rinsing them in distilled water, and the vascular specimens were then mounted on slides. The antibody used was rabbit anti-claudin 5 (Invitrogen Life Technologies, Carlsbad, CA, USA). To detect claudin-5, the vessels were incubated with Texas red-conjugated goat anti-rabbit antibodies (Santa Cruz, CA, USA). For the morphometric analysis, the density of the positive signal in the retina layer was determined using ImageJ software (NIH, Bethesda, MD, USA). Quantitative image analysis were independently performed by three researchers in a blind fashion and represented in a bar graph.

### 2.7. Western Blot Analysis

Retinal cytosolic proteins were extracted after homogenization in RIPA lysis buffer (Pierce Biotechnology, IL, USA). The proteins (20 µg) were separated using SDS-polyacrylamide gel electrophoresis, and then transferred to PVDF membranes (BioRad, Hercules, CA, USA). The membranes were blocked with 5% BSA in Tris-buffered saline containing Tween 20 (TBS-T) and washed three times with TBS-T. After an overnight incubation with anti-HMGB1 (Abcam, Cambridge, MA, USA) and anti β-actin antibodies (Sigma, MO, USA), the membranes were washed three times with TBS-T (10 min each), and immunoblots were developed using HRP-conjugated secondary antibodies and a chemiluminescence detection reagent (Amersham Bioscience, Piscataway, NJ, USA). The protein expression levels were determined by analyzing the signals captured on the PVDF membranes using an image analyzer (Las-3000, Fuji Photo, Tokyo, Japan). Quantitative image analysis were independently performed by three researchers in a blind fashion and represented in a bar graph.

### 2.8. Measurement of NF-κB Activity

Nuclear extracts from the retinal tissue were generated according to the manufacturer’s instructions (NE-PER nuclear and cytoplasmic extraction reagents, Thermo Scientific, IL, USA). Electrophoretic mobility shift assays (EMSAs) were performed by incubating 15 μg of the nuclear extract proteins in IRDye 700-labeled NF-κB oligonucleotide (LI-COR, Lincoln, NE, USA) that was dissolved in binding buffer (100 mM Tris, 500 mM KCl, and 10 mM DTT, pH 7.5) or with an unlabeled probe to study cold competition. The protein complexes that included the NF-κB oligonucleotide were separated on 4% non-denaturing polyacrylamide gels. Images of the gels images were captured and quantified using a LI-COR Odyssey infrared laser imaging system (LI-COR, Lincoln, NE, USA). Quantitative image analysis were independently performed by three researchers in a blind fashion and represented in a bar graph.

### 2.9. Chromatin Immunoprecipitation Assay

A chromatin immunoprecipitation (ChIP) assay was performed to analyze *in vivo* interactions between NF-κB and the corresponding *cis*-acting element in the RAGE promoter using a ChIP assay kit (Upstate Biotechnology, New York, NY, USA) in accordance with the manufacturer’s instructions. Soluble chromatin was prepared from retinal tissues. Chromatin was immunoprecipitated using anti-NF-κB p65 antibodies (2 µg each, Santa Cruz Biotechnology, CA, USA), and the negative control was rabbit IgG. The extracted DNA was used as a template for reverse transcription polymerase chain reaction (PCR) using the primer sets for the rat RAGE promoter regions containing the NF-κB response element (−720 to −441). The following primer sequences were used: forward, 5′-CCCGGCCCTGACTAAGCAGT-3′, and reverse, 5′-CCACGGCCTGGAACCCTTA-3′. One percent of the precipitated chromatin was assayed to verify equal loading. Quantitative image analysis were independently performed by three researchers in a blind fashion using Image J software (NIH, Bethesda, MD, USA) and represented in a bar graph.

### 2.10. Enzymatic Linked Immunosorbent Assay

To investigate the inhibitory effect of PCE on HMGB1/RAGE ligand/receptor binding, ligand binding was examined using *in vitro* sandwich ELISA. In total, 50 µL of a 40 µg dose of recombinant human HMGB1 (R & D Systems, MN, USA) was pre-coated and incubated on a microplate for 24 h at 4 °C and was then washed with PBS (pH 7.4). The sample was then blocked with 100 μL of CAS blocking solution (Life Technologies, Carlsbad, CA, USA) for 30 min at 37 °C and subsequently washed with PBS. The sample was then added to 50 µL of a 2.5 µg dose of recombinant human (rh) RAGE/FC chimera that was labeled with peroxidase (Dojindo, Kumanoto, Japan), and 50 µL of a serial dilution of the PCE mixture was placed in a microplate that was pre-coated with rh HMGB1. The samples were incubated for 2 h at 37 °C and then washed with PBS. A 3,3′,5,5′-Tetramethylbenzidine (TMB) chromogen solution was used as the substrate for the horseradish peroxidase. After the reaction was terminated with stop buffer (0.1 M H_2_SO_4_), and a yellow reaction product formed, and its absorbance was measured at 450 nm using a microtiter plate reader (Bio-Tek, Winooski, VT, USA). The inhibition of HMGB1 ligand receptor binding was expressed as a percentage decrease in the optical density (OD_450_ nm). We calculated the IC_50_ concentration (µg/mL) as a 50% inhibition of HMGB1/RAGE ligand/binding. Inhibitory activity was expressed as the mean ± S.D. of quadruplicate experiments. The IC_50_ value was calculated from the dose inhibition curve.

### 2.11. Statistical Analysis

The data are presented as the mean ± SEM and were calculated using analysis of variance followed by one-way analysis (ANOVA). The Tukey test was performed to evaluate differences using *Prism 6.0* software (GraphPad, San Diego, CA, USA), and *p* < 0.05 was considered significant.

## 3. Results

### 3.1. Analysis of PCE by HPLC

**Figure 1:**
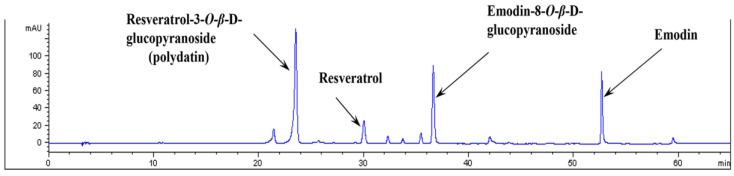
High performance liquid chromatography analysis. A qualitative analysis was performed to detect the fingerprint of PCE using high-performance liquid chromatography (HPLC). The compounds were identified by their peak retention times relative to reference substances.

A high-performance liquid chromatographic (HPLC) method was applied to the qualitatively analyze of the 100% EtOH extract of *P. cuspidatum*. We identified four marker compounds, including resveratrol-3-*O*-β-d-glucopyranoside (polydatin), resveratrol, emodin-5-*O*-β-d-glucopyranoside, and emodin, in the PCE by comparing the retention times with those of the four compounds. As shown in Figure 1, four compounds were selectively separated and identified in the HPLC chromatogram.

### 3.2. Blood Glucose Levels and Body Weight

Table 1 shows the physical characteristics at the beginning and end of the experiment. By the end of the experiment, the diabetic rats were hyperglycemic compared to the normal rats, and the diabetic rats exhibited lower body weights than the normal rats (*p* < 0.01). The blood glucose levels in the PCE-treated diabetic rats tended to be lower than those in the STZ-induced diabetic rats. However, no significant differences in blood glucose levels or weight gain were observed between the STZ-induced diabetic rats and the PCE-treated diabetic rats (Table 1).

### 3.3. Effect of PCE on HMGB1 Expression in the Retina

**Figure 2:**
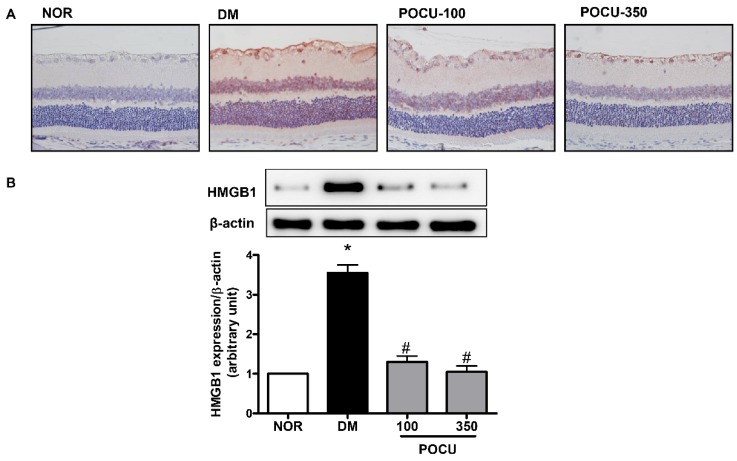
The effect of PCE on HMGB1 expression in the retina. (**A**) HMGB1 staining in the retina; (**B**) Western blot analysis of HMGB1 expression in a cytosolic extract of retinal tissue obtained from each group. Representative histological sections from a normal rat (NOR), an STZ-induced diabetic rat (DM), a DM treated with 100 mg/kg PCE (PCE-100), and a DM treated with 350 mg/kg PCE (PCE-350). The nuclei were counterstained using hematoxylin (**blue**). Original magnifications: ×400. All data are expressed as the mean ± SEM (*n* = 8). * *p* < 0.01 *vs.* the NOR group and # *p* < 0.01 *vs.* the DM group.

To investigate the pathogenic functions of HMGB1 in diabetic retinopathy, we initially used immunohistochemistry to determine whether the HMGB1 protein is expressed in the retina. HMGB1 has been recognized as a proangiogenic and pro-inflammatory factor, and upregulated HMGB1 expression in the retina has been reported [3]. We found that while HMGB1 was expressed at low levels in the ganglion cell layer of a normal retina, HMGB1 was highly expressed in the nuclei and diffusely expressed in the cytoplasm in the diabetic retinas, and HMGB1 expression in the retina was primarily localized within ganglion cells and the inner nuclear layer (Figure 2A). Additionally, western blot analysis showed that HMGB1 expression level was significantly higher in the STZ-induced diabetic retinal tissues than in the normal retinal tissues (Figure 2B). However, the increased HMGB1 expression in the diabetic rats was markedly decreased in the diabetic rats that were treated with PCE. This result shows that PCE treatment might have affected the level of HMGB1 expression in diabetic rat retinas.

### 3.4. Effect of PCE on RAGE Expression in the Retina

**Figure 3:**
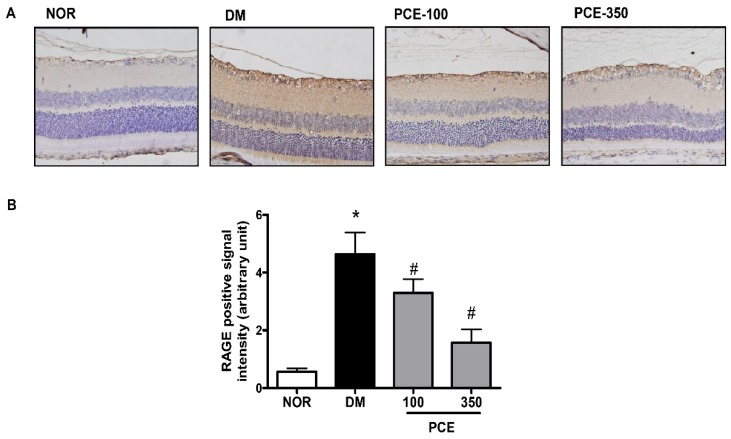
The effect of PCE on RAGE expression in the retina. (**A**) RAGE staining in the retina; (**B**) Morphometric analysis of the RAGE-positive signal density in the retina in each group. Representative histological sections from a normal rat (NOR), an STZ-induced diabetic rat (DM), a DM treated with 100 mg/kg PCE (PCE-100), and a DM treated with 350 mg/kg PCE (PCE-350). The nuclei were counterstained using hematoxylin (**blue**). Original magnifications: ×400. All data are expressed as the mean ± SEM (*n* = 8). * *p* < 0.05 *vs.* the NOR group and # *p* < 0.05 *vs.* the DM group.

When the rats were 24 weeks old, immunohistochemistry was performed on the diabetic rat retinas using an anti-RAGE antibody. The RAGE expression level was markedly higher in the STZ-induced diabetic rat retinas than in the normal rat retinas. This increase in the RAGE protein positive signal was significantly decreased in the PCE-treated diabetic rat retinas (Figure 3).

### 3.5. Effect of PCE on the Activation of NF-κB in the Retina

**Figure 4:**
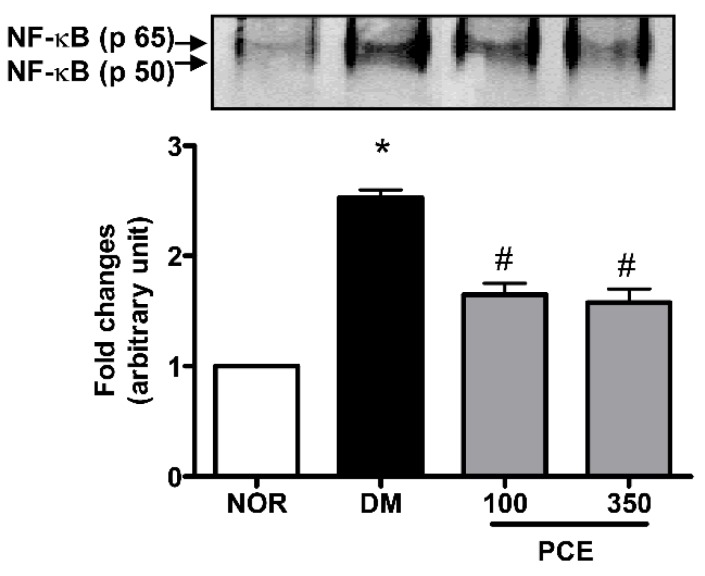
The effect of PCE on NF-κB activation in the retina. The DNA-binding activity of NF-κB was measured using EMSA. Representative protein expression in a normal rat (NOR), an STZ-induced diabetic rat (DM), a DM treated with 100 mg/kg PCE (PCE-100), and a DM treated with 350 mg/kg PCE (PCE-350). All data are expressed as the mean ± SEM (*n* = 8). * *p* < 0.01 *vs.* the NOR group and # *p* < 0.01 *vs.* the DM group.

We investigated whether the downstream activity of RAGE is mechanistically involves in the NF-κB signal pathway. The NF-κB DNA-binding activity of nuclear proteins that were isolated from retinal tissues was analyzed using EMSA, and our data indicated that NF-κB activity was markedly upregulated in the STZ-induced diabetic rats compared to the levels in the normal rats (*p* < 0.01). However, the treating the STZ-induced diabetic rats with PCE significantly decreased the induction of NF-κB activity (Figure 4).

### 3.6. Effect of PCE on the Activation of NF-κB in RAGE mRNA Expression

**Figure 5:**
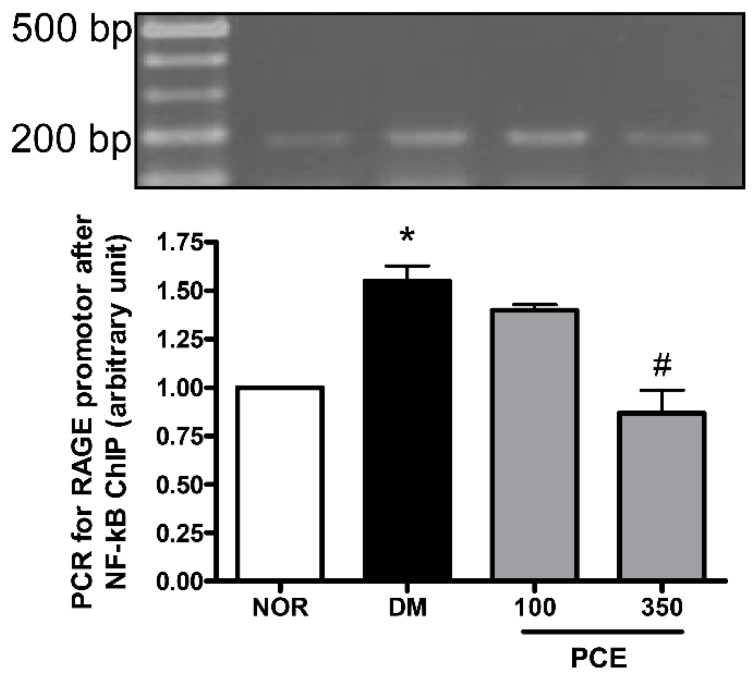
The effect of NF-κB on RAGE promoter expression in the retina. ChIP was performed using an anti-NF-κB p65 antibody, and the RAGE promoter was amplified using RT-PCR. The RT-PCR products from a normal rat (NOR), an STZ-induced diabetic rat (DM), a DM treated with 100 mg/kg PCE (PCE-100), and a DM treated with 350 mg/kg PCE (PCE-350) are shown. The PCR product size was 190 bp. The values in the bar graphs represent the mean ± SEM (*n* = 8). * *p* < 0.01 *vs.* the NOR group and # *p* < 0.01 *vs.* the DM group.

To evaluate the role of NF-κB in the induction of RAGE mRNA expression in diabetic retinas, the binding of NF-κB p65 to the RAGE promoter was analyzed using ChIP assays. We observed increased binding of this transcription factor to the RAGE promoter in STZ-induced diabetic rats. However, treating the diabetic rats with PCE significantly decreased the binding of NF-κB p65. These data strongly indicate that retinal RAGE expression is regulated by NF-κB and that PCE treatment exerts an inhibitory effect on RAGE expression by down-regulation of NF-κB (Figure 5).

### 3.7. Effect of PCE on HMGB1 Ligand to Receptor Binding

**Figure 6:**
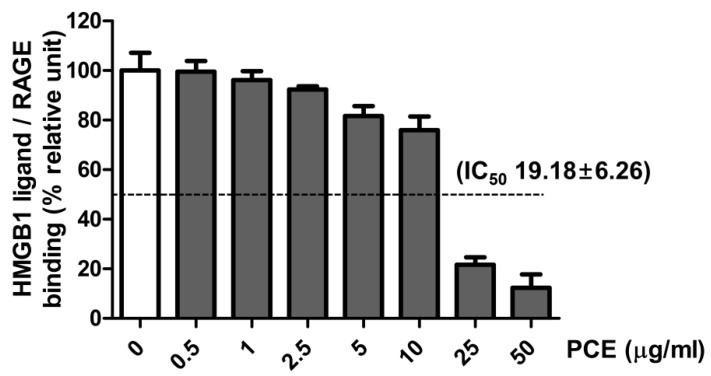
Effect of PCE on the binding of the HMGB1 ligand to its receptor (RAGE). The experiments were performed in quadruplicate. All data are expressed as the mean ± SEM.

To investigate whether PCE inhibits the binding of the HMGB1 ligand to its receptor (RAGE), a sandwich ELISA assay was performed. PCE exhibited an inhibitory effect on the binding of the HMGB1 ligand to its receptor (IC_50_ 19.18 ± 6.26 µg/mL) (Figure 6).

### 3.8. Effect of PCE on Diabetes-Induced Vascular Hyperpermeability

**Figure 7:**
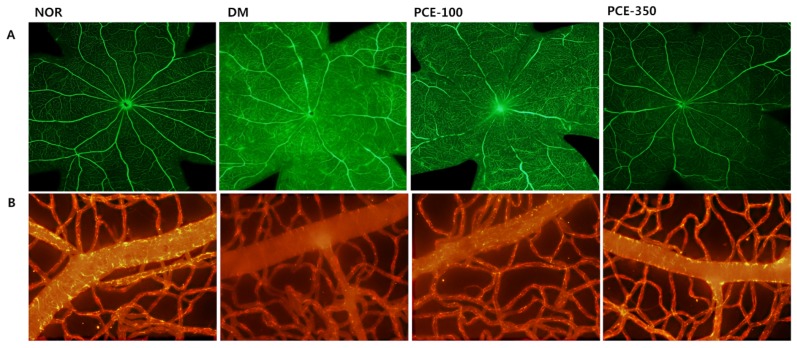
Effect of PCE on diabetes-induced retina vascular permeability. (**A**) Retinal vascular permeability in flat-mounted retinas was analyzed following FITC-BSA injections; and (**B**) immunofluorescence staining for the tight junction marker claudin-5 (Texas-red) in retinal vessels obtained from each group. Representative micrograph images of four independent experiments are shown at ×400 magnification. Representative histological sections from a normal rat (NOR), an STZ-induced diabetic rat (DM), a DM treated with 100 mg/kg PCE (PCE-100), and a DM treated with 350 mg/kg PCE (PCE-350) are shown.

To investigate the effects of PCE on the vascular functions in diabetic rats, BRB permeability and the loss of the tight junctions were measured following fluorescein isothiocyanate labelled bovine serum albumin (FITC-BSA) injection and immunofluorescence staining. The loss of the tight junctions increased retinal vascular permeability, and the fluorescence intensity was enhanced by dye (FITC-green) leakage throughout the entire retina in the STZ-induced diabetic rats. This leakage was markedly inhibited by treatment with PCE (Figure 7A). In normal retinas, claudin-5 was strongly detected on retinal vessels (Texas-red). The intensity of the signal corresponding to claudin-5 expression was reduced in the STZ-induced diabetic rat retinas compared to those in the normal rats. However, claudin-5 staining in the retinal vessels of the PCE-treated STZ-induced rats was markedly stronger than that observed in the STZ-induced diabetic rats (Figure 7B).

## 4. Discussion

Inflammation is now recognized as an important player in the pathogenesis of diabetic retinopathy, and a number of studies have been designed to determine whether blocking specific inflammatory molecules can be beneficial in diabetic retinopathy. In addition, inflammation is a cause of retinal vascular leakage in diabetic retinopathy [16]. HMGB1 is a multifunctional protein, and extracellular HMGB1 has been demonstrated to act as a pro-inflammatory factor in diabetic retinopathy. HMGB1 signals through its receptor, RAGE, which leads to the activation of the NF-κB signaling pathway in a diabetic retinal microvascular environment [5]. Furthermore, Zhang *et al.* reported that RAGE knockout prevented AGEs-induced microvascular hyperpermeability *in vivo* [17]. Thus, HMGB1 might induce retinal vascular leakage through the RAGE signal pathway. Although our data do not provide concrete evidence for a correlation between HMGB1 and vascular leakage in STZ- induced diabetic rats, this study shows that HMGB1 may induce retinal vascular leakage in part via its interaction with RAGE. In addition, the present study suggests that PCE prevents diabetes-induced retinal vascular hyperpermeability by attenuating the HMGB1 signaling pathway via the downregulation of the RAGE-mediated activation of NF-κB.

Hyperglycemia is an important causal factor that underlies the development of diabetic retinopathy. A type 1 diabetes mellitus (T1DM) animal model has often been used to study the mechanisms involved in diabetic complications behind the actions of anti-diabetes drugs independent of their glucose-lowering effects. Metformin and dipeptidyl peptidase IV (DPP4) inhibitors, such as vildagliptin, sitagliptin, and alogliptin, which are well-known anti-diabetes drugs for type 2 diabetes mellitus (T2DM), had no effect on body weight or blood glucose in the T1DM animal models [18,19,20,21]. Therefore, the purpose of this study was also to evaluate the effect of PCE on diabetic retinopathy in a model without a blood glucose reduction. In our study, PCE appeared to have a protective effect on the vasculature independent of glycemic control and body weight.

Diabetic retinopathy begins as a low-grade chronic inflammatory disease [1]. The activation of HMGB1/RAGE is an important signaling pathway that is involved in the initiation of the pro-inflammatory pathways that play important roles in diabetes-induced retinopathy [2]. A previous study demonstrated that the binding of HMGB1 to the RAGE receptor activates the NF-κB signaling pathways, which mediates the production of a variety of pro-inflammatory mediators, including HMGB1 and RAGE themselves [22]. Furthermore, it has been reported that the intravitreal injection of HMGB1 into normal rats upregulated the expression level of RAGE, NF-κB, and cytokines in the retina [2]. Consistent with the results in these studies, our data showed that the total and cytoplasmic HMGB1 expression levels were markedly elevated in STZ-induced diabetic rat retinal tissues. In addition, our data show that the treatment with PCE inhibited the HMGB1 signaling pathway in STZ-induced diabetic rats. HMGB1 has been shown to activate inflammatory signal pathways during diabetic microvascular complications and to disrupt the retinal vascular barrier [23]. One of the major ligands for RAGE is HMGB1, which is increased in the diabetic retinas [24,25,26]. In a previous study, when glycyrrhizin (a HMGB1 inhibitor) was administered in diabetic rats, the activation of NF-κB was attenuated, and tight junction protein expression was downregulated [17]. In addition, we performed an assay to determine whether PCE directly inhibits the binding of HMGB1 to its receptor, RAGE. As shown in Fig 6, PCE had an inhibitory effect against the HMGB1 ligand binding to RAGE, with an IC_50_ value of 19.18 ± 6.26 (µg/mL). These results suggest that PCE directly blocked the binding of HMGB1 to RAGE and indicate that PCE exerts a beneficial effect by preventing retinal vascular inflammation in diabetic retinopathy.

The formation and maintenance of the blood-retinal barrier (BRB) is critical to normal retinal vascular function in diabetic retinopathy. In addition, the breakdown of the BRB, an early feature of diabetic retinopathy, results in vascular leakage [27]. Inflammation is correlated with retinal vessel occlusion and capillary dropout, and retinal vessel occlusion and degeneration is a typical feature of diabetic retinopathy [16]. Claudin-5 is a marker of tight junctions that is necessary to preserve the vascular barrier to the entry of small molecules into the brain [28]. It also plays a similar role in the BRB [29]. In the present study, fluorescein angiography demonstrated that PCE markedly inhibited fluorescein leakage, suggesting that PCE might prevent the breakdown of the BRB. Furthermore, we found that claudin-5 was markedly reduced in diabetic retinal microvessels. However, these phenomena were completely prevented by treatment with PCE. These data strongly suggest that PCE has the ability to protect against diabetes-induced retinal vascular hyperpermeability.

Some popular traditional herbs have been used for generations as food flavoring agents and medicinal treatments in Asian countries. *P. cuspidatum* extract has been used to treat inflammation, infection, skin burns, and hyperlipidemia [13]. Many studies have demonstrated that the ethanol extract of the *P. cuspidatum* root exerts many pharmacological effects, including anti-inflammatory, anti-diabetes, and lipid regulatory effects. In an animal model, Bralley *et al.* demonstrated that PCE reduced edemas and inhibited leukocyte infiltration to a greater extent than indomethacin, which is a potent anti-inflammatory drug [30]. In addition, previous studies have shown that an extract of *P. cuspidatum* radix exerted an inhibitory effect on RAGE and VEGF expression in diabetic nephropathy [15] and exhibited strong anti-lipase activity [31]. These investigations have supported the notion that PCE has anti-inflammatory, anti-lipase, and anti-diabetic effects. Moreover, the current study has also shown that PCE ameliorated the expression level of HMGB1 in diabetic rat retinal tissues and inhibited the binding of NF-κB to the RAGE promoter in diabetic retinas. These results suggest that PCE has a considerable anti-inflammatory activity during the progression of diabetic retinopathy.

In the present study, resveratrol and emodin and the derivatives of each compound, including resveratrol-3-*O*-β-d-glucopyranoside (polydatin) and emodin-8-*O*-β-d-glucopyranoside, were detected in an HPLC analysis. *P. cuspidatum* is a nutraceutical substance that is used for medical purpose because of the consistently high concentration of its major compounds, resveratrol and polydatin [13]. *P. cuspidatum* extracts also contains other compounds, such as emodin that display anti-inflammatory activity [32]. Resveratrol and polydatin have been shown to act as polyphenolic compounds to perform several biological functions, including anti-inflammatory and anti-oxidant activities. Xu *et al.* demonstrated that resveratrol prevented diabetes-induced renal inflammation by inhibiting the Akt/NF-κB pathway in an animal model [33]. Recently, resveratrol was shown to effectively reduce the expression of HMGB1, RAGE, and various cytokines, including TNF-α and IL-4, in inflamed skin caused by atopic dermatitis [18]. Zheng *et al.* suggested that the effect of resveratrol on vascular inflammatory injuries was associated with the downregulation of the NF-κB signaling pathway [34]. In addition, Polydatin has been demonstrated to have anti-inflammatory and anti-diabetic effects by inhibiting the activation of the NF-κB signaling pathway *in vivo* [35]. The potential efficacy of emodin for treating various inflammatory diseases is based on its inhibition of the activation of NF-κB, which is involved in the transcription of various pro-inflammatory genes known to be involved in disease progression [36]. Moreover, Lee *et al.* first demonstrated that emodin suppresses the release of HMGB1 and the activation of NF-κB by HMGB1 in *in vivo* and *in vitro* experiments [37]. Emodin effectively inhibited NF-κB activation and IκB degradation and decreased the gene expression of cell surface adhesion proteins in vascular endothelial cells [38]. Furthermore, emodin-8-*O*-β-d-glucopyranoside prevented inflammation by increasing the total antioxidant capacity of cells after cerebral ischemia [39]. In the present study, we show that PCE effectively prevented the diabetes-induced upregulation of the expression of HMGB1 and RAGE and NF-κB in the retina. These results indicate that PCE may exert synergistic effects with other chemical compounds, such as resveratrol, polydatin, and emodin. Although the major compounds in PCE include resveratrol, polydatin, and emodin, we did not assess the individual effects of these compounds on diabetic retinopathy. Nevertheless, our study strongly demonstrates that PCE may be able to prevent diabetes-induced retinal vascular hyperpermeability via its inhibitory effect on the upregulation of HMGB1 by the RAGE-mediated activation of NF-κB.

Our results demonstrate that the oral administration of PCE ameliorates hyperglycemia-induced retinal dysfunction independently of effect on blood glucose control and body weight in STZ-induced diabetic rats. The ability of PCE to prevent diabetes-induced retinal vascular hyperpermeability may be the result of the effects of its components compounds, which include resveratrol, polydatin, and emodin. Therefore, our study indicates that therapies that target retinal vascular dysfunction-mediated inflammation using PCE may significantly help to suppress the development of diabetic retinopathy.

## Figures and Tables

**Table 1:** Blood glucose levels and body weight.

	Time	NOR	DM	PCE-100	PCE-350
Non-fasting BG (mg/dL)	Initial	145.7 ± 9.5	351.5 ± 101.4 *	349.5 ± 90.2	347.0 ± 86.8
	End	145.9 ± 19.1	592.8 ± 17.6 *	520.4 ± 77.3	446.0 ± 132.0
Fasting BG (mg/dL)	Initial	77.8 ± 8.4	292.3 ± 76.9 *	288.6 ± 74.7	288.5 ± 74.8
	End	162.1 ± 11.0	587.3 ± 43.5 *	530.3 ± 58.1	474.2 ± 88.4
Body weight (g)	Initial	307.3 ± 17.5	226.6 ± 18.7 *	224.2 ± 20.6	219.7 ± 16.2
	End	475.9 ± 10.7	208.5 ± 12.1 *	205.5 ± 23.1	206.0 ± 16.4

NOR, normal rat; DM, STZ-induced diabetic rats; PCE-100, DM treated with 100 mg/kg PCE; PCE-350, DM treated with 350 mg/kg PCE. All data are expressed as the mean ± SEM (*n* = 8). * *p* < 0.01 *vs.* the NOR group*.*

## References

[1] Adamis A.P. (2002). Is diabetic retinopathy an inflammatory disease?. Br. J. Ophthalmol..

[2] Santos A.R., Dvoriantchikova G., Li Y., Mohammad G., Abu El-Asrar A.M., Wen R., Ivanov D. (2014). Cellular mechanisms of high mobility group 1 (HMGB-1) protein action in the diabetic retinopathy. PLoS ONE.

[3] Voll R.E., Urbonaviciute V., Herrmann M., Kalden J.R. (2008). High mobility group box 1 in the pathogenesis of inflammatory and autoimmune diseases. Isr. Med. Assoc. J..

[4] Lee Y.M., Kim J., Jo K., Shin S.D., Kim C.S., Sohn E.J., Kim S.G., Kim J.S. (2013). Ethyl pyruvate inhibits retinal pathogenic neovascularization by downregulating HMGB1 expression. J. Diabetes Res..

[5] Mohammad G., Siddiquei M.M., Othman A., Al-Shabrawey M., Abu El-Asrar A.M. (2013). High-mobility group box-1 protein activates inflammatory signaling pathway components and disrupts retinal vascular-barrier in the diabetic retina. Exp. Eye Res..

[6] Luan Z.G., Zhang H., Yang P.T., Ma X.C., Zhang C., Guo R.X. (2010). HMGB1 activates nuclear factor-kappaB signaling by RAGE and increases the production of TNF-alpha in human umbilical vein endothelial cells. Immunobiology.

[7] El-Asrar A.M., Nawaz M.I., Kangave D., Geboes K., Ola M.S. (2011). High-mobility group box-1 and biomarkers of inflammation in the vitreous from patients with proliferative diabetic retinopathy. Mol. Vis..

[8] Kim J., Sohn E., Kim C.S., Jo K., Kim J.S. (2011). The role of high-mobility group box-1 protein in the development of diabetic nephropathy. Am. J. Nephrol..

[9] Lim B.O., Lee J.H., Ko N.Y., Mun S.H., Kim J.W., Kim do K., Kim J.D., Kim B.K., Kim H.S., Her E. (2007). Polygoni cuspidati radix inhibits the activation of Syk kinase in mast cells for antiallergic activity. Exp. Biol. Med..

[10] Peng W., Qin R., Li X., Zhou H. (2013). Botany, phytochemistry, pharmacology, and potential application of *Polygonum cuspidatum* Sieb.et Zucc.: A review. J. Ethnopharmacol..

[11] Kirino A., Takasuka Y., Nidhi A., Kawabe S., Yamashita H., Kimoto M., Ito H., Tsuji H. (2012). Analysis and functionality of major polypenolic components of *Polugonum cuspidatum* (Itadory). J. Nutr. Sci. Vitaminol..

[12] Bob F., Philippe S., Flaws B., Sionneau P. (1897). The treatment of modern medical diseases with Chinese Medicine. Text Book and Clinal Manual.

[13] Han J.H., Koh W., Lee H.J., Lee E.O., Lee S.J., Khilc J.H., Tae K.J., Jeonga S.J., Kim S.H. (2012). Analgesic and anti-inflammatory effects of ethyl acetate fraction of *Polygonum cuspidatum* in experimental animals. Immunopharmacol. Immunotoxicol..

[14] Zhang H., Yu C.H., Jiang Y.P., Peng C., He K., Tang J.Y., Xin H.L. (2012). Protective effects of polydatin from *Polygonum cuspidatum* against carbon tetrachloride-induced liver injury in mice. PLoS ONE.

[15] Fei H.H., Xiao Z.D., Gao W. (2008). Effects of giant knotweed rhizome medicine on the expressions of RAGE and VEGF of rats with diabetic nephropathy. J. Shandong Univ. Health Sci..

[16] Zhang W., Liu H., Rojas M., Caldwell R.W., Caldwell R.B. (2011). Anti-inflammatory therapy for diabetic retinopathy. Immunotherapy.

[17] Zhang W., Xu Q., Wu J., Zhou X., Weng J., Xu J., Wang W., Huang Q., Guo X. (2015). Role of Src in vascular hyperpermeability induced by advanced glycation end products. Sci. Rep..

[18] Tan Z., Xu Z., Gui Q., Wu W., Yang Y. (2014). Gliquidone *versus* metformin: Differential effects on aorta in streptozotocin induced diabetic rats. Chin. Med. J..

[19] Liu W.J., Xie S.H., Liu Y.N., Kim W., Jin H.Y., Park S.K., Shao Y.M., Park T.S. (2012). Dipeptidyl peptidase IV inhibitor attenuates kidney injury in streptozotocin-induced diabetic rats. J. Pharmacol. Exp. Ther..

[20] Gonçalves A., Marques C., Leal E., Ribeiro C.F., Reis F., Ambrósio A.F., Fernandes R. (2014). Dipeptidyl peptidase-IV inhibition prevents blood-retinal barrier breakdown, inflammation and neuronal cell death in the retina of type 1 diabetic rats. Biochim. Biophys. Acta.

[21] Davidson E.P., Coppey L.J., Dake B., Yorek M.A. (2011). Treatment of streptozotocin-induced diabetic rats with alogliptin: Effect on vascular and neural complications. Exp. Diabetes Res..

[22] Karuppagounder V., Arumugam S., Thandavarayan R.A., Pitchaimani V., Sreedhar R., Afrin R., Harima M., Suzuki H., Nomoto M., Miyashita S. (2014). Resveratrol attenuates HMGB1 signaling and inflammation in house dust mite-induced atopic dermatitis in mice. Int. Immunopharmacol..

[23] Manigrasso M.B., Juranek J., Ramasamy R., Schmidt A.M. (2014). Unlocking the biology of RAGE in diabetic microvascular complications. Trends Endocrinol. Metab..

[24] McVicar C.M., Ward M., Colhoun L.M., Guduric-Fuchs J., Bierhaus A., Fleming T., Schlotterer A., Kolibabka M., Hammes H.P., Chen M. (2015). Role of the receptor for advanced glycation endproducts (RAGE) in retinal vasodegenerative pathology during diabetes in mice. Diabetologia.

[25] Chen M., Curtis T.M., Stitt A.W. (2013). Advanced glycation end products and diabetic retinopathy. Curr. Med. Chem..

[26] Zong H., Ward M., Stitt A.W. (2011). AGEs, RAGE, and diabetic retinopathy. Curr. Diabetes Rep..

[27] Navaratna D., McGuire P.G., Menicucci G., Das A. (2007). Proteolytic degradation of VE-cadherin alters the blood-retinal barrier in diabetes. Diabetes.

[28] Nitta T., Hata M., Gotoh S., Seo Y., Sasaki H., Hashimoto N., Furuse M., Tsukita S. (2003). Size-selective loosening of the blood-brain barrier in claudin-5-deficient mice. J. Cell Biol..

[29] Leal E.C., Martins J., Voabil P., Liberal J., Chiavaroli C., Bauer J., Cunha-Vaz J., Ambrósio A.F. (2010). Calcium dobesilate inhibits the alterations in tight junction proteins and leukocyte adhesion to retinal endothelial cells induced by diabetes. Diabetes.

[30] Bralley E.E., Greenspan P., Hargrove J.L., Wicker L., Hartle D.K. (2008). Topical anti-inflammatory activity of *Polygonum cuspidatum* extract in the TPA model of mouse ear inflammation. J. Inflamm..

[31] Kim Y.S., Lee Y.M., Kim J.H., Kim J.S. (2013). *Polygonum cuspidatum* inhibits pancreatic lipase activity and adipogenesis via attenuation of lipid accumulation. BMC Complement. Altern. Med..

[32] Lee G., Choi T.W., Kim C., Nam D., Lee S.G., Jang H.J., Lee J.H., Um J.Y., Jung S.H., Shim B.S. (2013). Anti-inflammatory activities of *Reynoutria elliptica* through suppression of mitogen-activated protein kinases and nuclear factor-kappaB activation pathways. Immunopharmacol. Immunotoxicol..

[33] Xu F., Wang Y., Cui W., Yuan H., Sun J., Wu M., Guo Q., Kong L., Wu H., Miao L. (2014). Resveratrol prevention of diabetic nephropathy is associated with the suppression of renal inflammation and mesangial cell proliferation: Possible roles of Akt/NF-kappab pathway. Int. J. Endocrinol..

[34] Zheng X., Zhu S., Chang S., Cao Y., Dong J., Li J., Long R., Zhou Y. (2013). Protective effects of chronic resveratrol treatment on vascular inflammatory injury in streptozotocin-induced type 2 diabetic rats: Role of NF-kappa B signaling. Eur. J. Pharmacol..

[35] Chen L., Lan Z., Lin Q., Mi X., He Y., Wei L., Lin Y., Zhang Y., Deng X. (2013). Polydatin ameliorates renal injury by attenuating oxidative stress-related inflammatory responses in fructose-induced urate nephropathic mice. Food Chem. Toxicol..

[36] Xie X., Peng J., Huang K., Huang J., Shen X., Liu P., Huang H. (2012). Polydatin ameliorates experimental diabetes-induced fibronectin through inhibiting the activation of NF-kappaB signaling pathway in rat glomerular mesangial cells. Mol. Cell. Endocrinol..

[37] Shrimali D., Shanmugam M.K., Kumar A.P., Zhang J., Tan B.K., Ahn K.S., Sethi G. (2013). Targeted abrogation of diverse signal transduction cascades by emodin for the treatment of inflammatory disorders and cancer. Cancer Lett..

[38] Lee W., Ku S.K., Kim T.H., Bae J.S. (2013). Emodin-6-*O*-beta-d-glucoside inhibits HMGB1-induced inflammatory responses *in vitro* and *in vivo*. Food Chem. Toxicol..

[39] Kumar A., Dhawan S., Aggarwal B.B. (1998). Emodin (3-methyl-1,6,8-trihydroxyanthraquinone) inhibits TNF-induced NF-kappaB activation, IkappaB degradation, and expression of cell surface adhesion proteins in human vascular endothelial cells. Oncogene.

